# From Militant Voices to Militant Irony: Examining Identity, Memory and Conflict in the Basque Country

**DOI:** 10.5964/ejop.v13i3.1324

**Published:** 2017-08-31

**Authors:** Ignacio Brescó de Luna

**Affiliations:** a Department of Communication and Psychology, Aalborg University, Aalborg, Denmark; Webster University Geneva, Geneva, Switzerland; University of Neuchâtel, Neuchâtel, Switzerland

**Keywords:** identity, memory, conflicts, narratives, irony

## Abstract

Collective memory and identity so often go hand in hand with conflicts. Alongside the use of violence, conflicts unfold against the backdrop of different narratives about the past through which groups constantly remind themselves of the supposed origin of the conflict, and consequently, what position individuals are expected to take as members of the group. Narratives – as symbolic tools for interpreting the past and the present, as well as happenings that have yet to occur – simultaneously underpin, and are underpinned by, the position held by each warring faction. Drawing on previous works, this paper compares different versions of the 2016 truce period in the Basque Country stemming from three subjects identified, to varying degrees, with the main political actors involved in that conflict. These three cases have been selected from a total of 16 participants who were asked to define the Basque conflict and to provide an account of the 2006 truce period by using 23 documents taken from different Spanish newspapers. On the one hand, the results show two narratives reproducing the versions of two of the main political actors involved in the conflict, and on the other hand, a narrative characterized by a more personal and ironic appropriation of those versions. Results are discussed vis-à-vis the use of irony in history teaching in increasingly plural societies.

Identity and memory are virtually inseparable concepts. On the one hand, it is a known fact that memory, understood as being the reconstruction of the past, is a deciding factor in respect of both individual and collective identity. According to certain authors closely associated with the narrative turn that took place in the Human Sciences (Polkinghorne, 1988), the existence of a narrative is crucial in that it relates the past to the present on the basis of the same entity involved in the account, thus enabling us to believe in the permanence of that same entity throughout time, be it *self* (Ricoeur, 1991) or an *imagined community* such as nations (Anderson, 1983). On the other hand, we are also aware, from the pioneering work of Halbwachs (1950/1980) and Bartlett (1932), that the way in which we remember our past is strongly influenced by the groups in which we live and by which we identify ourselves. Memory is social from the outset, insofar as groups provide us with both symbolic resources – language or narrative structures (Wertsch, 2002) – and motifs for constructing and sharing our memories. Furthermore, since infancy we are exposed to a constant barrage of accounts of the past, whether in informal contexts such as family or formal contexts such as school. These are narratives which, despite occurring prior to our own existence, end up forming part of our past and therefore our identity.

We can thus say that our personal identity is irredeemably linked to collective identity, since by appropriating the group’s past accounts, we become emotionally involved in them and absorb the values, achievements, grievances and collective claims conveyed by those accounts. In fact, this socially shared sense of the past is what often leads us to identify with the positions defended by individuals who claim to represent certain groups or collectives, and to assume them as our own, in the first person plural. This way of transmitting the past and making it available and familiar to individuals has led some authors (Bar-Tal, 2014; Liu & Hilton, 2005) to study collective memory through the social representations theory (Moscovici, 1984). From this perspective, memory is considered as something dynamic, “actively engaged, socially and materially situated, reconstructive and oriented to the future” (Wagoner, 2015, p. 143). This dynamic implies, on the one hand, a process of *objectification*, by which the group’s historical past is conveyed through a range of stories, images, rituals, monuments etc., and on the other hand, a process of *anchoring*, whereby the group’s representation of the past becomes a framework against which to interpret the present and imagine the future.

Collective memory and identity so often go hand in hand with conflicts to the point that they seamlessly feed into each other. Thus, on one hand, conflicts deeply mark groups’ collective memory and identity, whether in the form of deeds to be collectively remembered and celebrated or grievances and affronts not to be forgotten. On the other hand, memory and identity are elements that underlie many conflicts, insofar as certain ways of enhancing a common sense of belonging are built upon groups’ old – or recent – scars, grievances, resentments and hatreds, thus providing a rationale through which conflicts may be fuelled, reignited and perpetuated. In this sense, alongside the use of violence, conflicts unfold against the backdrop of different narratives about the past through which groups constantly remind themselves of the supposed origin of the conflict, and consequently, what position individuals are expected to take as members of the group. Thus, narratives – as symbolic tools for interpreting the past and the present, as well as happenings that have yet to occur – simultaneously underpin, and are underpinned by, the position held by each warring faction (see Harré & van Langenhove, 1999). This gives rise to a symbolic and argumentative context (Brescó & Wagoner, 2016) saturated by different partisan narratives, these in turn being the symbolic tools or mediational means (Wertsch, 1991) by which people come to give sense to conflicts and build a position according to the group with which they identify.

In such divided and multivoiced contexts, the possible standpoints on the conflict are pretty much constrained by warring factions’ discourses and voices, which individuals – identified with different groups – tend to appropriate and make their own. This would explain why in conflicts deemed as intractable (Bar-Tal, 2013) – where groups are somehow locked in their own positions and versions of the conflict (see Nicholson, 2016) – new favourable scenarios for reconciliation are usually perceived and anchored in light of the old narratives and ways of representing the conflict. In such cases, in which a window for peace seems to emerge, alternative approaches tend to be overshadowed by partisan ways of interpreting reality.

## Identity, Memory, and Conflict in the Basque Country

The Basque Country conflict in Spain – now on the way to be resolved – is a clear example of this^i^. After fifty years of violence and social unrest, the armed group ETA (acronym for *Euzkadi ta Azcatasuna* or *Basque Country and Freedom* in English) announced a permanent ceasefire in March 2006. This announcement was surrounded by controversy since the very beginning due to the different ways in which the ceasefire was interpreted by the main political actors involved in the conflict. ETA considered the ceasefire as the first step to negotiate the independence of the Basque Country from the Spanish State. The Spanish Government (headed at that time by the Socialist José Luís Rodríguez Zapatero) deemed the ceasefire as an opportunity to initiate a peace process which could lead to a negotiated solution to the conflict. By its part, the main opposition party (the right wing People’s Party) considered the ceasefire as a “truce-trap” and accused the Government of having secret deals with the terrorists and surrendering the country to ETA. In December that year, ETA planted a bomb at Madrid airport, leaving two people dead. That attack – justified by ETA as a response to the Government’s passivity during the truce period – was the tragic outcome of nine months of political unrest during which the main actors involved resorted to different accounts in order to justify their own positions vis-à-vis a process understood as a “democratic process” according to Batasuna, a “peace process” from the Government’s point of view and a “trick process” by the People’s Party.

Thus, following the bombing attack at Madrid airport, diverse ways of understanding that process were consolidated by means of various opposing accounts; accounts that also acted as tools by which people could interpret, recall and draw conclusions from the ceasefire according to their identification with the main figures involved. This is not to say that agency was vanished amidst warring factions’ narratives and voices. Agency lays on the irreducible tension between those meditational means provided by a particular socio-cultural setting – in this case, the public narratives about the ceasefire period – and the way these are used by individuals as a resource in remembering (Wertsch, 2002). In Bakhtinian terms this implies different forms of multivoiced authoring (Bakhtin, 1981; see also Wertsch & O’Connor, 1994) in that different voices (in this case, those pertaining to the main figures involved in the conflict) would be appropriated and adapted to individuals’ own intentions in different specific contexts. Such a personal appropriation of social discourses may take different degrees of agency and authorship, ranging from reproduction and acceptance, at one end of the continuum, to entire rejection on the other (Brescó, 2016; Wertsch & O’Connor, 1994). However, in polarised contexts saturated with partisan discourses, it is often the case that the rejection of one implies embracing some other. It is in these particular contexts – such as the Basque conflict – where personal appropriation of these discourses in the form of irony or satire becomes a way of resisting – and mocking – the warring factions’ militant discourses saturating the public sphere.

### Case Study: Remembering the 2006 Truce Period in the Basque Country

Drawing on previous works (Brescó, 2009, 2016), this section aims to compare different versions of the abovementioned truce period stemming from three subjects identified to varying extents with the main political actors involved in that Basque conflict^ii^. These three cases form part of a wider study in which a total of 16 undergraduate students – from the University of the Basque Country and the Autonomous University of Madrid – were asked to define the Basque conflict and to provide an account of the 2006 ceasefire period by using 23 short documents extracted from TV and different Spanish newspapers^iii^. The documents used for this study – arranged in chronological order – consisted of five pictures, ten broadsheet headings and eight brief extracts of statements delivered by some political actors during the truce period (see examples of each type in Table 1). Participants were allowed to use the documents in any way they wished (e.g., only using those supporting their views on the truce period, omitting those others they deemed unimportant or at odds with their viewpoints, and adding whatever extra information they considered appropriate).

**Table 1:** Examples of the Different Type of Documents Provided to Participants

Type of document	Example
Broadsheet heading	“More detentions, reports of torture and prohibitions of Batasuna’s demonstrations” (*Gara*, pro Batasuna newspaper) “ETA steals 300 guns with large amounts of ammunition in the southeast of France” (*ABC*, right-wing newspaper)
Political statement	“The People’s Party’s cooperation is key to achieving the end of violence” (Spanish Prime Minister José Luís Rodríguez Zapatero) “Pretty soon the government will go back to its bad ways and will negotiate with ETA again” (Ángel Acebes, People’s Party member)
Picture	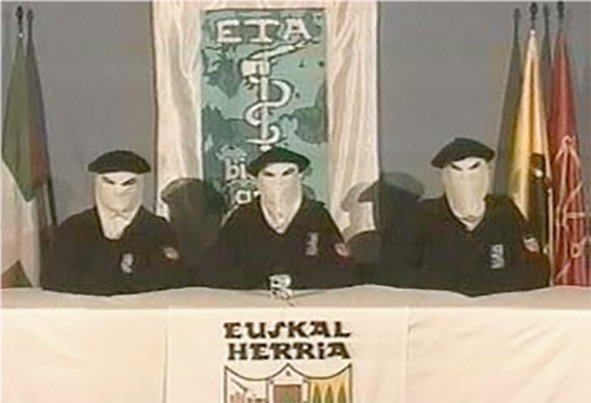 Members of ETA announcing the cease-fire (*El País*, centre-left wing newspaper)

Analysis of the accounts on the peace process yielded by participants focused on how their respective thematization and positioning vis-à-vis the conflict mediated the way this event was reconstructed – viz, which documents were used or ignored by each participant and what meaning and function these documents acquired within each account. Of particular interest here is showing how these three subjects appropriate and use different narratives of the truce period in light of their respective identification with the main figures involved. Along these lines, results include, on the one hand, two versions reproducing the story-line provided by two of these figures^iv^, and on the other and, a version characterized by a more personal, critical and ironic appropriation of these narratives.

*Participant 1: Green^v^* is an 18-year-old male who studies psychology at the University of the Basque Country and who identifies with Batasuna. The following is Green’s definition of the Basque conflict and his account of the ceasefire period:

Definition of the Basque conflict: *“It is a conflict between an oppressed nation and two oppressor states (Spain and France) which refuse to recognize the right all democratic states have: the right to self-determination. This situation has led to an armed conflict*.”

Account of the ceasefire period: *“ETA declares a truce and ceases all its actions. The Government says that it is willing to meet with ETA. Batasuna expresses its willingness to negotiate the future of the Basque Country. The Spanish state keeps imprisoning, torturing and oppressing the Basque People, especially Batasuna and its supporters. Given the course of events and the Government’s inability to move forward, ETA decides to send out a warning to the Government by planting a bomb at Madrid airport. The Government doesn’t react and the truce comes to an end. Then, ETA returns to its armed struggle*.”

Green clearly takes Batasuna’s position and makes it his own, thus defining the conflict as one caused by the oppression of the Basque People by both the Spanish and French state. Building up on such position, the participant justifies and delegitimizes the actions of the main figures involved in the ceasefire period. According to Green’s version, despite ETA’s good intentions, the Spanish Government failed in its democratic duty by not listening to the Basque people, thereby causing ETA to exercise its right to resume the armed struggle. It is also worth noting that in order to support this version, the participant omitted all documents (included among the material handed out to the subjects) referring to ETA’s violent activities during the ceasefire, and instead used the news article from *Gara* – a newspaper with close ties to Batasuna – which spoke of arrests and torture vis-à-vis Batasuna and its supporters. Here, it is the Government that is responsible for the truce’s failure by not responding to ETA’s warning in the form of a bomb attack, this being conceived as a form of communication.

*Participant 2: Blue* is an 18-year-old female who studies psychology in Madrid and sympathizes with the right-wing People’s Party. She sees the conflict and the ceasefire period as follows:

Definition of the Basque conflict: *“There is a group of people from that region who don’t feel Spanish so they use violence*.”

Account of the ceasefire period: *“Thanks to a series of secret agreements between ETA and the Socialist Party, with many concessions made by the latter, a truce was achieved. During the supposed truce period, the Government was completely willing to hold talks with the terrorists while they kept on committing terrorist acts. Thousands of Spaniards marched, demanding that Zapatero stop yielding to ETA’s claims. In turn, the People’s Party split with the Government due to Zapatero’s erroneous strategy. This event ended with the terrorist attack on Madrid Airport, which caused two casualties (this is, in fact, the only way ETA understands dialogue). After this attack, we are still supposed to believe that the Government has dropped negotiations with ETA.”*

Blue considers the violence employed by a supposed anti-Spanish faction as being the origin of the Basque conflict, thus somehow assuming a connection between not feeling Spanish and the use of violence. In analysing Blue’s version, we can see how this participant takes People’s Party stance on the truce period and makes it her own. In accordance to that, Blue tends to use those newspapers closer to that party (see endnote iii). Thus, unlike Green’s version, she assesses the truce in quite a negative light, considering it as a “plot” between the Socialist Government and ETA, pretty much in line with those right-wing newspapers and politicians who supported the conspiracy theory along the peace process. For example, Blue’s words echo the statement by a People’s Party member – included in the material – whereby ETA’s ceasefire announcement was linked to Zapatero’s supposed previous concessions to that group. Along these lines, Blue’s account refers to one of the pictures provided featuring numerous people demonstrating against government’s peace-making process. Blue also includes in her account some of the news referred to some of the alleged ETA’s terrorist activities during the truce period. Additionally, Blue explicitly refers to the terrorist attack and the two resulting casualties – an attacked depicted in one of the pictures provided to the participants. Interestingly enough, her remark on this tragic outcome – which she describes as ETA’s only way of understanding dialogue – echoes Green’s version in which the attack was conceived as a form of communication, in this case as a mere warning. All in all, Blue´s version of the events would prove the uselessness of dialogue with the terrorists in line with the People’s Party position against the government’s attempt to reach a peace agreement with ETA. In light of this, the position taken by the People’s Party throughout the peace process is no longer a failure, but a patriotic duty against a preceding immoral agreement.

*Participant 3: Gray* is a 23-year-old male who studies psychology in Madrid. He sympathises with the Socialist Party and *Izquierda Unida* (United Left), a party to the left of the former which also supported the peace-making process. Gray’s view on both the conflict and the truce period is as follows:

Definition of the Basque conflict: *“This is a somewhat fictitious conflict. I don’t think that it’s about the Basque people’s claim for independence, or at least, it’s not just about that. I believe that both sides feed off each other’s positions and live off keeping the conflict alive to some extent.”*

Account of the ceasefire period: *“On the 22^nd^ of March three gentlemen wearing hoods and fancy dress appear on TV announcing a truce. They pledge not to kill for a certain period of time whereas the Government undertakes nobody knows what. Everybody is very happy about what is deemed the beginning of a peace process and because the end of violence is thought to be near. Immediately afterwards, all the political and media machinery is set in motion. The politicians start to calculate every move in terms of electioneering benefits. On the one hand, the People’s Party, in order to discredit the Socialists, insinuates that ETA’s and the Government’s interests are basically the same. On the other hand, the Socialist Party does everything in its power to prevent the process from getting out of hand, trying to please everybody with promises. As for the Basque extreme nationalists, they try to appear as the champions of peace in order to obtain greater support among the people and thus reinforce their presence in the institutions. The constant attacks and innuendos launched by the People’s Party end up undermining Zapatero’s popularity, thus leading the Government to adopt a tougher line against ETA. At the same time, the members of ETA who are not interested in giving up the struggle manage to impose their strategy which finally results in the bomb attack at Madrid airport. With this tragic episode, both the peace process and the cheap farce set up around it come to an end”.*

Gray’s stance is removed from the position of the main political actors as he sees the conflict as something fictitious, fuelled by the actors themselves. From this standpoint, the claims deriving from each actor’s position become meaningless insofar as they would constitute a resource for nourishing a conflict that all sides wish to keep alight. Such a critical distance is reflected in the way the truce period is narrated. Thus, from the very first sentence (*On the 22^nd^ of March three gentlemen wearing hoods and fancy dress appear on TV announcing a truce*), Gray makes clear his resistance to take seriously what the actors involved in the truce period claim to be doing. This ironic stance on what is considered a fictitious conflict is further reinforced by his explicitly likening the peace-process to a “*cheap farce*”. Along these lines, the whole episode is narrated as if it were a play, one that starts off with the appearance on the stage (in this case, on television) of the three members of ETA announcing the ceasefire and the activation of all the political and media machinery, which continues to operate until the bombing attack on Madrid airport. This way of reconstructing the ceasefire period moreover underscores the fictitious nature of the position of the actors involved, actors whose ‘performance’ is aimed more at making their respective audiences happy – i.e., not losing popularity among their voters and supporters – than at having their claims satisfied – be it achieving independence, reaching an agreement through dialogue or defending the unity of the Spanish state.

### Militant Voices and Militant Irony in the Basque Conflict

As we can see in these three examples, participants Green and Blue clearly identify themselves with certain political actors (ETA and the People’s Party, respectively), thus assuming and to some extent reproducing their corresponding versions of the truce period, including the claims, criticisms and justifications contained in such versions. As a result, we have two militant accounts in which each participant seems to accept and adopt the voice of one of those actors and make it their own. From a Bakhtinian perspective, we could say that the actors’ voices speak through the participants’ accounts, or in other words, that participants have been to some degree talked—or ventriloquized—by those actors’ voices.

Contrary to Green’s and Blue’s acceptance of such voices, Gray’s critical stance on both ETA and People’s Party positions is reflected through an ironic, even satirical, narrative style by which this participant criticises the absurd logic that characterises these actors’ conduct and by extension the whole conflict at large. This is carried out by means of a certain way of using the voices of the political actors themselves in order to highlight their absurdity during the truce period. This resource, linked to irony, is close to the Bakhtinian concept of ‘double-voicedness’, “refer[ed] to the use of someone else’s words in order to express one’s own intentions and meanings that are hostile to others’ words” (Marková, 2003, p. 63). We can see examples of this at the beginning of Gray’s account, when he speaks ironically about both ETA’s ceasefire (*“they pledge not to kill for a certain period of time whereas the Government undertakes nobody knows what”*) and the general optimism and faith in relation to the truce and the end of the conflict (*“everybody is very happy about what is deemed the beginning of a peace process and because the end of violence is thought to be near”*).

In Gray´s case, we find a greater degree of agency in reconstructing the truce period compared to the cases of Green and Blue. Thus, whereas in the latter cases the participants’ words expressed the view of the main political actors on the truce period, in Gray’s case the words of those political actors are used to express the participant’s more personal view of it. In this regard, Gray’s satirical and distant stance is not incompatible with the adoption of his own positioning on the episode in question. As Frye (1957) points out in his work on *Tropics of Discourse*, “satire is militant irony: its moral norms are relatively clear, and it assumes standards against which the grotesque and absurd are measured” (p. 223). This moral dimension related to the use of tropes and genres is further developed by the philosopher of history Hayden White who argues that the narrative forms used in reconstructing the past inevitably convey a moral content (White, 1986). In the particular case of irony, this author considers this genre as a meta-trope, a trope related to self-consciousness in the use of language when talking about the past. In White’s own words, “[irony] represents a stage of consciousness in which the problematical nature of language itself has become recognized” (White, 1973, p. 37).

## Memory, Identity and History Teaching: Towards an Ironic Citizenship?

Identity and memory are elements that can easily be found in many conflicts. Groups transmit and use narratives about the past in order to underpin their identity as well as their respective position within conflicts. These narratives act as mediational tools through which the members of the group not only reconstruct the past – viz., how the conflict originated – but also anchor and give meaning to present events as well as the conflict’s future horizon. Transmission of these narratives takes place in different contexts, such as the family and school. As for the latter, according to Carretero (2011), history teaching continues to focus on “intimate emotional adherence to national identity symbols and narratives – in detriment to critical thinking” (p. 38). According to Misztal (2003), this identity-based conveyance of historical narratives serves to both legitimize certain political structures and claim historically marginalized identities. In both cases, the objective lies in instilling loyalty towards the different collectives, leading individuals to assume as their own the collectives’ past and future, their defeats and victories, their heroes and enemies. As Liu and Hilton (2005) state, social representations of the past have a mobilizing power as they outline a trajectory telling “us who we are, where we came from and where we should be going” (p. 537).

This leads us to highlight the role of imagination, or more specifically the *politics of imagination* (Bottici & Challand, 2011), both regarding collective identity – how our identity is imagined in opposition to a certain alterity (see Glăveanu & de Saint Laurent, 2015) – and collective memory – how the imagined collective future affects the way of remembering the past (see de Saint Laurent, Obradovic, & Carriere, in press). In fact, the role of collective memory – in this case, regarding conflicts – can be understood as a way of reconstructing the past in light of different imagined futures in order to foster current actions, thus proleptically guiding the present towards certain political goals (see the notion of prolepsis applied to collective memory in Brescó, 2017). From this point of view, it can be argued that collective memory conveys a script with guidelines for action as well as for interpreting the actions of other political actors involved in the conflict; a script that, in providing the story-line of the conflict, not only describes what happened in the past and how is the state of things in the present, but it also prescribes what lines of actions should be taken in the future. In making these scripts their own, individuals run the risk of becoming trapped in certain positions in the conflict, thus becoming actors of a ready-made story-line. This is usually the case whenever warring factions’ discourses saturate the public sphere, thus making it difficult for alternative versions and voices to be articulated, let alone heard. In such cases, in the absence of any alternative available discourse, the use of irony constitutes a way in which to resist the official versions of the conflict. As we have seen in the previous section, the theatrical metaphor used by the third participant in our study (Gray), is not only a resource to mock official and partisan versions of the truce period; it also denotes a greater degree of agency through which that participant can denaturalize and distance himself from those versions, thus gaining more authorship over his own way of recounting this episode.

Identity and memory are highly flammable elements whose misuse in certain contexts may make them ignite into conflict. However, at the same time they can be important elements for an open and reflective citizenry. Memory does not just keep hatreds alight. By looking at the past we can gain knowledge about our mistakes and wrongdoings, victims can be remembered and compensated, and more reflective ways of dealing with history can be promoted in order to avoid new conflicts in the future (Wagoner & Brescó, 2016). In the same vein, the presence of different identities, far from being a threatening reality, constitutes an opportunity to reconstruct the group’s inherited narratives about the past, which thus opens the door to rethinking and generating more complex and flexible identities and positions that are open to change and diversity (Rosa & González, 2012). History teaching, in this sense, is called upon to contribute to this endeavour by denaturalizing historical narratives and encouraging the democratic participation of citizens in the public affairs of plural societies (see Rosa & Brescó, 2017). In discussing the role of history teaching in an increasingly globalized world, authors such as Rorty (1989) and Turner (2002) see irony as a way in which to foster a sceptical attitude towards traditional national histories so that more open and cosmopolitan views can be promoted (see Smith, 2007 for a discussion on this matter).

Irony can indeed be a means by which to prevent individuals from naturalizing certain narratives transmitted through the group, narratives which may hinder dialogue and reconciliation with others. In line with Egan’s (1997) notion of *ironic understandin*g regarding history teaching, Blanco and Rosa (1997) float the idea that “perhaps it would not be a bad goal to look for an ironic citizenship, but an irony based upon reflection and informed dialogue, not cynicism” (p. 15). Maybe irony is not enough, but it is probably a necessary element in order to endow people with more agency so that they can gain perspective on identity, memory and conflicts.
